# 

**Published:** 1892-02

**Authors:** 


### Southern Medical Record

A Monthly Journal of Medicine and Surgery.

VOL. XXII. ATLANTA, GA., FEBRUARY, 1892. NO. 2.

EDITORS:

A. W. GRIGGS, M. D. WILLIS F. WESTMORELAND, M. D.

J. McFADDEN GASTON, M D. DAN. H. HOWELL, M D., Bus. Mgr.

Entered at the Postoffice at Atlanta, Ga., as Second-Class Matter. Index to Advertisements on Page 27

Postell & Sexton, Publishers. 47 S. Broad, Atlanta. Ga.

